# Impact of Early MPO-ANCA Positivity on Unique Clinical Features in Korean Patients with EGPA: A Single-Centre Cohort Study

**DOI:** 10.3390/medicina61061088

**Published:** 2025-06-13

**Authors:** Oh Chan Kwon, Jang Woo Ha, Min-Chan Park, Yong-Beom Park, Sang-Won Lee

**Affiliations:** 1 Division of Rheumatology, Department of Internal Medicine, Gangnam Severance Hospital, Yonsei University College of Medicine, Seoul 06273, Republic of Korea; ockwon@yuhs.ac (O.C.K.);; 2 Division of Rheumatology, Department of Internal Medicine, Yongin Severance Hospital, Yonsei University College of Medicine, Yongin 16995, Republic of Korea; 3 Division of Rheumatology, Department of Internal Medicine, Yonsei University College of Medicine, Seoul 03722, Republic of Korea; 4 Institute for Immunology and Immunological Diseases, Yonsei University College of Medicine, 50-1 Yonsei-ro, Seodaemun-gu, Seoul 03722, Republic of Korea

**Keywords:** anti-neutrophil cytoplasmic antibody, vasculitis, myeloperoxidase, clinical, feature

## Abstract

*Objectives*: Previous studies have suggested differences in vasculitic and eosinophilic phenotypes based on anti-neutrophil cytoplasmic antibody (ANCA) positivity in eosinophilic granulomatosis with polyangiitis (EGPA). However, their relevance under the 2022 American College of Rheumatology (ACR)/European Alliance of Associations for Rheumatology (EULAR) classification criteria remains unclear. We aimed to evaluate the clinical features and outcomes of EGPA according to myeloperoxidase (MPO)-ANCA status in a Korean cohort. *Methods*: We conducted a retrospective cohort study that included 57 patients with EGPA without proteinase 3-ANCA positivity who fulfilled the 2022 ACR/EULAR classification criteria. Patients were classified into MPO-ANCA-positive (n = 25) and MPO-ANCA-negative (n = 32) groups. Clinical manifestations, laboratory findings, and outcomes, including all-cause mortality, relapse, end-stage kidney disease (ESKD), cerebrovascular accident (CVA), and acute coronary syndrome (ACS), were compared between the two groups. *Results*: MPO-ANCA-positive patients exhibited higher Five-Factor Scores (1.0 [0.0–1.0] vs. 0.0 [0.0–1.0], *p* = 0.038), lower Short Form 36 Physical Component Summary scores (35.0 [19.7–56.3] vs. 52.5 [43.5–69.7], *p* = 0.048), and elevated systemic inflammation markers (higher erythrocyte sedimentation rate: 58.0 [16.0–97.5] mm/hr vs. 25.5 [7.0–63.8] mm/hr, *p* = 0.026). Constitutional symptoms were more frequent among MPO-ANCA-positive patients (n = 14 [56.0%] vs. n = 3 [9.4%], *p* < 0.001), whereas no significant differences were found in vasculitic or eosinophilic manifestations. Kaplan–Meier analysis revealed no differences in the overall (*p* = 0.36), relapse-free (*p* = 0.80), ESKD-free (*p* = 0.87), CVA-free (*p* = 0.26), or ACS-free (*p* = 0.94) survival rates between the two groups. *Conclusions*: In Korean patients with EGPA classified under the 2022 ACR/EULAR classification criteria, MPO-ANCA positivity, as compared to ANCA-negative status, was associated with a higher disease burden and poorer quality of life but not with distinct vasculitic or eosinophilic manifestations and adverse outcomes.

## 1. Introduction

Eosinophilic granulomatosis with polyangiitis (EGPA) is a subtype of anti-neutrophil cytoplasmic antibody (ANCA)-associated vasculitis (AAV), characterised by late-onset asthma, peripheral eosinophilia, and necrotising vasculitis affecting small- to medium-sized vessels [1]. It is a rare disease with a global prevalence of 15.27 cases per 1,000,000 individuals [2]. ANCA positivity is observed in approximately 40% of patients with EGPA, with myeloperoxidase (MPO) being the predominant target antigen among these cases [3]. Several studies on European patients with EGPA have demonstrated that ANCA positivity is associated with distinct clinical manifestations: ANCA-positive patients more frequently exhibit vasculitic features, such as glomerulonephritis, peripheral neuropathy, and purpura, whereas ANCA-negative patients more commonly present with eosinophilic features, such as cardiomyopathy and gastroenteritis [4,5,6,7]. Similarly, a study from China reported that MPO-ANCA-positive patients exhibited a higher frequency of vasculitic features, including renal involvement and biopsy-proven vasculitis, whereas MPO-ANCA-negative patients more commonly exhibited eosinophilic features, such as cardiac involvement and asthma [8]. However, regarding the prognostic implications of ANCA status, studies have shown conflicting results: a French study reported a higher 10-year survival rate among ANCA-positive patients than among ANCA-negative patients (*p* = 0.02) [4], whereas a Chinese study demonstrated comparable survival rates between the two groups (*p* = 0.78) [8], warranting further investigation. Moreover, whether ANCA status is associated with the development of major comorbidities that are important causes of death in EGPA, such as acute coronary syndrome (ACS), cerebrovascular accidents (CVA), and end-stage kidney disease (ESKD), remains unclear [9].

In 2022, new classification criteria for EGPA were proposed by the American College of Rheumatology (ACR) and the European Alliance of Associations for Rheumatology (EULAR) [10] which were not applied in earlier studies [3,4,5,6,7]. The criteria do not include MPO-ANCA when considering the assignment of negative points solely to proteinase 3 (PR3)-ANCA positivity. The omission of MPO-ANCA from the classification criteria raises important questions regarding its diagnostic relevance and potential utility in predicting outcomes in clinical practice.

Therefore, we aimed to evaluate the differences in clinical manifestations and outcomes, including all-cause mortality, relapse, ESKD, CVA, and ACS, between MPO-ANCA-positive and MPO-ANCA-negative patients with EGPA, as defined by the 2022 ACR/EULAR classification criteria [9], in a Korean cohort.

## 2. Materials and Methods

### 2.1. Study Patients

We conducted a retrospective cohort study using data from the Severance Hospital ANCA-associated vasculitides (SHAVE) cohort, a prospective single-centre cohort of Korean patients with AAV. Comprehensive details of the SHAVE cohort have been published elsewhere [11]. From this cohort, we identified 63 patients who met the 2022 ACR/EULAR classification criteria for EGPA [10]. Six patients with PR3-ANCA positivity were excluded to avoid potential confounding arising from phenotypic differences associated with PR3-ANCA status [12]. The remaining 57 patients with EGPA without PR3-ANCA positivity were included in the analysis. According to MPO-ANCA positivity, patients were categorised into MPO-ANCA-positive (n = 25) and MPO-ANCA-negative (n = 32) groups.

This study was approved by the Institutional Review Board (IRB) of Severance Hospital (Seoul, Republic of Korea, IRB No. 4-2020-1071) and conducted in accordance with the Declaration of Helsinki. Given the retrospective design of the study and the use of anonymised patient data, the requirement for written informed consent was waived.

### 2.2. Clinical and Laboratory Data at EGPA Diagnosis

Clinical characteristics and laboratory findings at the time of EGPA diagnosis were obtained through a comprehensive review of electronic medical records. Demographic data, including age, sex, body mass index, and smoking history, were reviewed. Data on ANCA positivity and AAV-specific indices, such as the Birmingham Vasculitis Activity Score (BVAS), Five-Factor Score (FFS), and Short Form 36 (SF-36) Physical Component Summary (PCS) and Mental Component Summary (MCS), were collected. Clinical manifestations based on the 2022 ACR/EULAR classification criteria and the BVAS were also reviewed. Acute phase reactants (erythrocyte sedimentation rate [ESR] and C-reactive protein [CRP]) and laboratory data, including complete blood cell counts, fasting glucose, blood urea nitrogen, serum creatinine, total protein, and serum albumin, were obtained. Data on the presence of type 2 diabetes mellitus, hypertension, and dyslipidaemia were reviewed as comorbidities.

### 2.3. Medications and Outcomes

The use of glucocorticoids and immunosuppressants during the follow-up period was assessed as a binary variable (‘yes’ or ‘no’). The outcomes of interest were all-cause mortality, relapse, ESKD, CVA, and ACS. The follow-up period was defined as the period from EGPA diagnosis to the occurrence of the respective outcome in affected patients and from EGPA diagnosis to the last follow-up visit, up to June 2024, in those without the outcome.

### 2.4. Statistical Analyses

Continuous variables are reported as median (Q1–Q3 range), and categorical variables are reported as number (%). The normality of continuous variables was assessed using the Shapiro–Wilk test. For comparison between the MPO-ANCA-positive and -negative groups, the Mann–Whitney U test was used for continuous variables, while the chi-square test or Fisher’s exact test was used for categorical variables. The cumulative, relapse-free, ESKD-free, CVA-free, and ACS-free survival rates were analysed using Kaplan–Meier analysis. The log-rank test was employed to compare survival rates between the groups. A *p*-value of <0.05 was considered statistically significant. All analyses were conducted using SPSS software (version 28.0; IBM Corporation, Armonk, NY, USA).

## 3. Results

### 3.1. Baseline Characteristics of the Study Patients

The baseline characteristics of the 57 patients with EGPA without PR3-ANCA positivity are shown in Table 1. The median age was 53.0 (42.5–63.5) years, and 19 (33.3%) patients were male. MPO-ANCA (or perinuclear-ANCA) positivity was observed in 25 (43.9%) patients. The median values of the BVAS, FFS, SF-36 PCS, and SF-36 MCS were 11.0 (8.0–17.0), 1.0 (0.0–1.0), 47.4 (31.1–65.4), and 54.1 (41.4–76.1), respectively. Regarding clinical manifestations, otorhinolaryngological involvement (82.5%) was the most common, followed by pulmonary (68.4%) and central and peripheral nervous system (59.6%) involvement. The median values of the ESR and CRP were 41.0 (10.5–68.0) mm/hr and 5.6 (1.1–22.0) mg/L, respectively. The median eosinophil count was 790.0 (300.0–4290.0)/mm^3^.

### 3.2. Comparison Between MPO-ANCA-Positive and MPO-ANCA-Negative Patients

Compared with MPO-ANCA-negative patients, MPO-ANCA-positive patients had a higher FFS (1.0 [0.0–1.0] vs. 0.0 [0.0–1.0], *p* = 0.038) and lower SF-36 PCS (35.0 [19.7–56.3] vs. 52.5 [43.5–69.7], *p* = 0.048) (Table 2). General manifestations, such as fever, malaise, myalgia, and arthralgia, were more common in MPO-ANCA-positive patients (56.0% vs. 9.4%, *p* < 0.001). However, the frequencies of vasculitic (cutaneous: *p* = 0.40; renal: *p* = 0.14; and central and peripheral nervous systems: *p* = 0.26) and eosinophilic manifestations (cardiac: *p* > 0.99; gastrointestinal: *p* > 0.99) did not differ between the two groups.

The ESR was significantly higher (58.0 [16.0–97.5] vs. 25.5 [7.0–63.8] mm/h, *p* = 0.026) in MPO-ANCA-positive patients. Haemoglobin (12.7 [11.7–13.8] vs. 13.7 [12.7–14.6] g/dL, *p* = 0.012) and serum albumin (3.7 [3.1–4.1] vs. 4.1 [3.5–4.4] g/dL, *p* = 0.042) levels were significantly lower in MPO-ANCA-positive patients.

A comparison of the medications used during follow-up between the groups is shown in Table 3. No significant differences in the use of glucocorticoids and immunosuppressants were observed between the two groups.

### 3.3. Comparison of Outcomes Between MPO-ANCA-Negative and -Positive Patients

During a median follow-up of 59.6 (28.6–110.5) months, the incidence of all-cause mortality did not differ between the MPO-ANCA-negative and -positive patients (0.0% vs. 4.0%, *p* = 0.44). Similarly, the incidence of other outcomes, including relapse (9.4% vs. 16.0%, *p* = 0.69), ESKD (3.1% vs. 4.0%, *p* > 0.99), CVA (0.0% vs. 4.0%, *p* = 0.44), and ACS (3.1% vs. 8.0%, *p* = 0.58), also did not differ between the two groups. A detailed comparison of outcomes between the two groups is summarised in Table 4.

**Figure 1:**
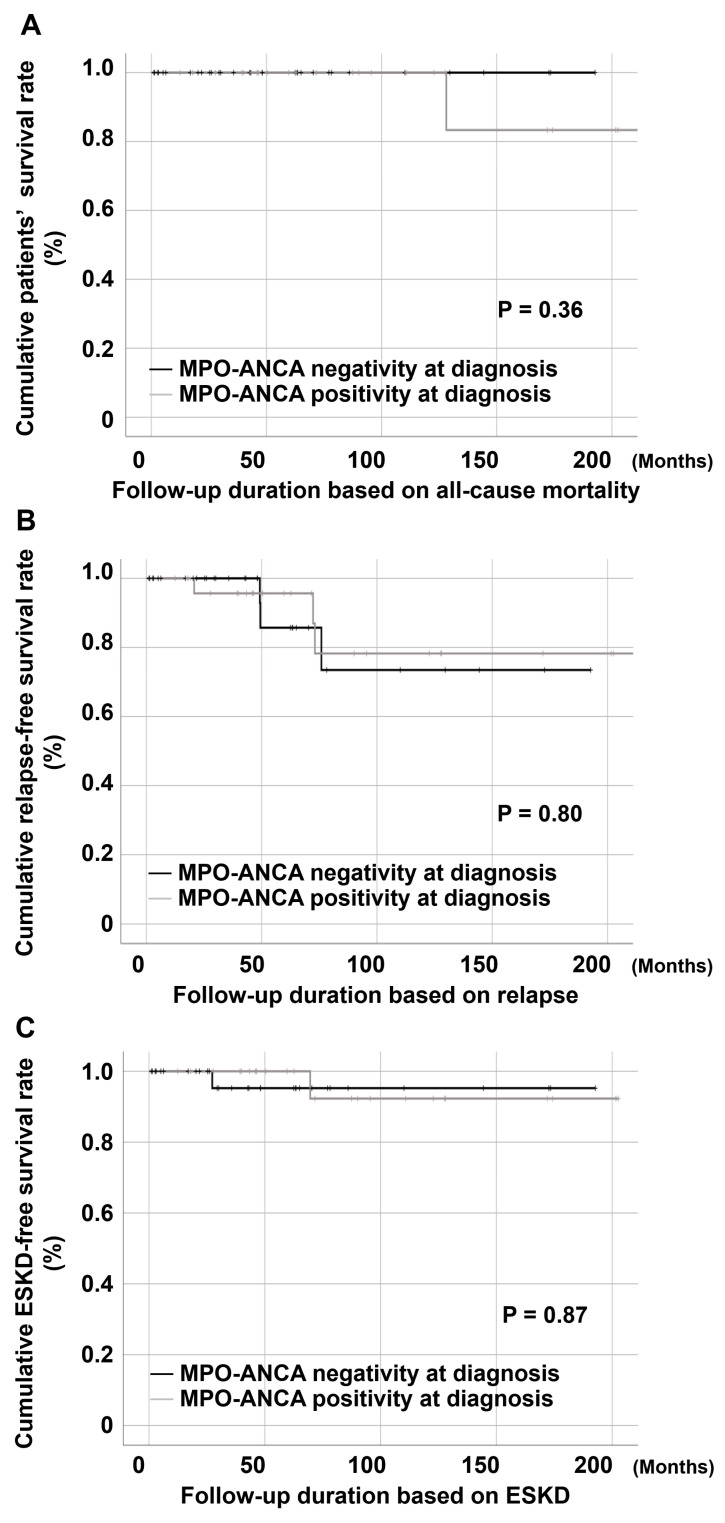

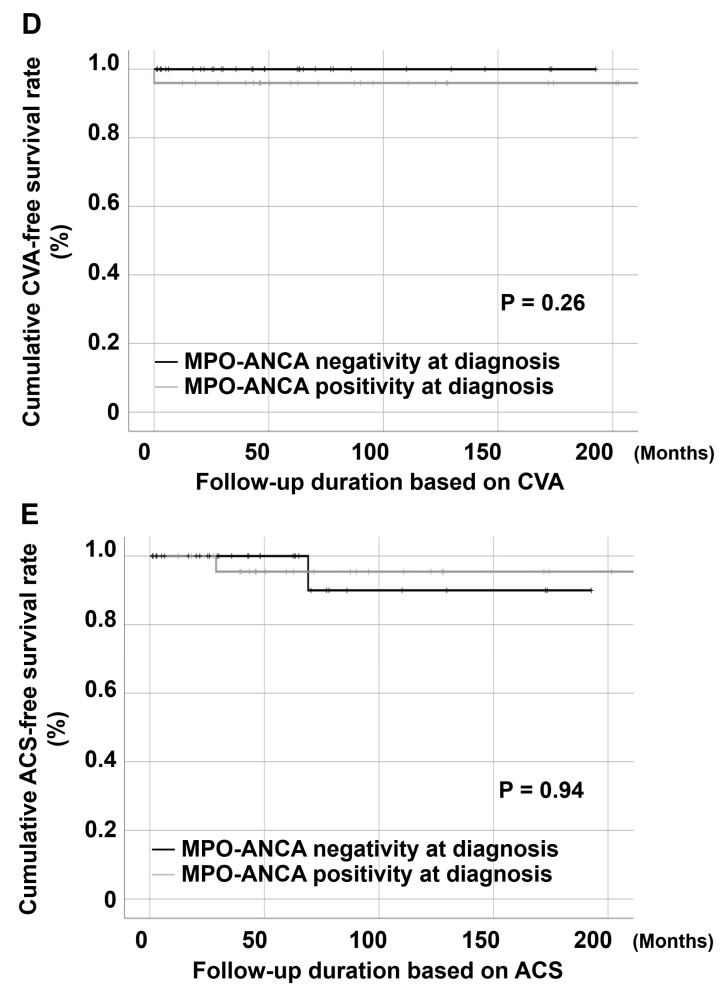
Kaplan-Meier analysis showing cumulative (**A**) survival rate, (**B**) relapse-free survival rate, (**C**) ESKD-free survival rate, (**D**) CVA-free survival rate, and (**E**) ACS-free survival rate according to MPO-ANCA status. ACS: acute coronary syndrome; ANCA: anti-neutrophil cytoplasmic antibody; CVA: cerebrovascular accident; ESKD: end-stage kidney disease; MPO: myeloperoxidase.

The results of the Kaplan–Meier analyses are shown in Figure 1. The cumulative (Figure 1A, *p* = 0.36), relapse-free (Figure 1B, *p* = 0.80), ESKD-free (Figure 1C, *p* = 0.87), CVA-free (Figure 1D, *p* = 0.26), and ACS-free (Figure 1E, *p* = 0.94) survival rates did not differ between the two groups.

## 4. Discussion

In this study, we investigated the differences in clinical features and outcomes of Korean patients with EGPA according to MPO-ANCA status. Among 57 patients with EGPA without PR3-ANCA positivity, we found that MPO-ANCA-positive patients exhibited distinct clinical and laboratory characteristics compared with MPO-ANCA-negative patients. Notably, constitutional symptoms, such as fever, malaise, myalgia, and arthralgia, were more frequent in MPO-ANCA-positive patients. Furthermore, higher FFS and lower SF-36 PCS values were observed in MPO-ANCA-positive patients. In addition, differences in laboratory markers of inflammation (higher ESR in MPO-ANCA-positive patients) were observed, suggesting a potential association between MPO-ANCA positivity and higher disease burden. However, cumulative survival, relapse-free survival, and major adverse outcomes, including ESKD-, CVA-, and ACS-free survival, were comparable between the two groups.

Our findings contrast with those of prior studies conducted in European [4,5,6,7] and Chinese [8] cohorts, which reported a dichotomy between vasculitic and eosinophilic manifestations based on the ANCA status. In these studies, MPO-ANCA-positive patients had higher frequencies of vasculitic manifestations (purpura, renal involvement, and peripheral neuropathy), whereas MPO-ANCA-negative patients more often exhibited eosinophilic manifestations (cardiac involvement and gastrointestinal symptoms) [4,5,6,7,8]. Our findings also differ from those of a recent study by Drynda et al., which used data from the POLVAS registry and reported a higher prevalence of cardiovascular manifestations in patients with ANCA-negative EGPA than in those with MPO-ANCA-positive EGPA [13]. However, in our cohort, vasculitic and eosinophilic features did not differ significantly between the two groups. Instead, only general manifestations were more frequent among MPO-ANCA-positive patients. A possible explanation for this discrepancy with previous findings may be the use of different classification criteria for patient selection. In earlier studies [4,6,7,8,13], patients with EGPA were selected according to the 1990 ACR classification criteria [14] and/or the Chapel Hill definition [15]. In contrast, the patients in our study were selected according to the 2022 ACR/EULAR classification criteria [10]. In the 2022 ACR/EULAR classification criteria, MPO-ANCA positivity has become a major determinant in the classification of MPA, being assigned a score of 6, which is sufficient on its own to classify a patient with biopsy-proven small- to medium-sized vasculitis as MPA, even in the absence of other relevant features [16]. Under the 2022 ACR/EULAR classification criteria [10,16], patients with MPO-ANCA positivity, asthma, mononeuritis multiplex, migratory pulmonary infiltrates, and biopsy-proven small- to medium-sized vasculitis with extravascular eosinophil infiltration but without eosinophilia exceeding 1000/mm^3^ would no longer be classified as having EGPA, as was possible under previous criteria, but would instead be reclassified as having MPA. As a result, MPO-ANCA-positive patients in previous studies may have included individuals who, under the 2022 ACR/EULAR classification criteria, would be reclassified as having MPA, a condition that is inherently more associated with vasculitic than eosinophilic features. If these patients were to be reclassified under the current criteria, the previously reported dichotomy between MPO-ANCA-positive and -negative EGPA might not be observed. This may explain the lack of distinct phenotypic separation in our cohort.

Instead of the dichotomy between vasculitic and eosinophilic manifestations according to MPO-ANCA positivity, we found that MPO-ANCA-positive patients have a significantly higher FFS than MPO-ANCA-negative patients. Given that the FFS includes age > 65 years, cardiac involvement, renal insufficiency, gastrointestinal involvement, and absence of ear, nose, and throat (ENT) involvement [17], the observed difference may be partly explained by the greater number of patients aged > 65 years, lower ENT involvement, and increased renal involvement in the MPO-ANCA-positive group, although these individual components did not reach statistical significance. Additionally, our study is the first to demonstrate that SF-36 PCS scores are significantly lower in MPO-ANCA-positive patients, indicating poorer health-related quality of life. This has not been addressed in previous studies and suggests that MPO-ANCA status may be associated with the quality of life of patients. The lower SF-36 PCS score in the MPO-ANCA-positive group may be attributable to the higher prevalence of organ involvement, which directly impairs physical functioning. In particular, pulmonary (59.4% vs. 80.0%, *p* = 0.10), renal (15.6% vs. 32.0%, *p* = 0.14), and central and peripheral nervous system (53.1% vs. 68.0%, *p* = 0.26) manifestations tended to be more frequent in this group, although statistical significance was not reached. We believe that these in total may account for the observed difference in SF-36 PCS scores between the two groups. Moreover, although the BVAS did not differ between the groups, MPO-ANCA-positive patients had a higher ESR, suggesting a higher systemic inflammatory burden. These findings imply that in patients with EGPA who are classified based on the 2022 ACR/EULAR classification criteria [10], MPO-ANCA positivity may be more closely associated with disease burden and health-related quality of life than with a dichotomous pattern of vasculitis- or eosinophil-specific manifestations. Importantly, our study is the first to investigate the clinical impact of MPO-ANCA status in EGPA using the 2022 classification criteria, providing novel insights that differ from previous findings (derived using older criteria). This updated framework allows for a more precise delineation of the clinical burden associated with MPO-ANCA positivity and may inform more individualised monitoring strategies in clinical practice.

We also analysed a comprehensive set of outcomes, including not only all-cause mortality and relapse but also ESKD, CVA, and ACS. No significant differences were observed in any of these outcomes between the MPO-ANCA-positive and -negative groups. Although ANCA-positive patients are theoretically more prone to cardiovascular and renal complications [18,19], our findings suggest that MPO-ANCA positivity may not independently predict poor outcomes in patients with EGPA. Furthermore, while MPO-ANCA-positive patients had higher FFS and lower SF-36 PCS scores, which are recognised as indicators of higher mortality in patients with AAV [17,20], these factors did not translate into higher mortality (i.e., lower survival rate) in our cohort. This discrepancy may be attributable to the small number of mortality events observed during the follow-up period, which likely limited the statistical power to detect significant differences. Over a median follow-up duration of approximately 60 months, no deaths were observed in the MPO-ANCA-negative group, and only one death occurred in the MPO-ANCA-positive group. Given the low event rate, the analysis may have been underpowered for detecting differences in the cumulative survival rate (Figure 1A, *p* = 0.36). Therefore, while our findings do not demonstrate a statistically significant association between MPO-ANCA positivity and mortality, the possibility of a prognostic impact cannot be entirely excluded and warrants further investigation in larger cohorts.

This study had some limitations. First, owing to the retrospective design of the study, the outcomes could have been affected by unmeasured confounders such as undocumented comorbid conditions. Second, the relatively small sample size may have reduced the statistical power to detect subtle differences in clinical features and outcomes. Furthermore, owing to the relatively small sample size and single-centre nature of the cohort, the findings may not be generalisable to the entire Korean population. Third, although our findings suggest an association between MPO-ANCA positivity and greater disease burden, the underlying immunopathogenic mechanisms linking ANCA status to clinical severity were not explored in this study. Future studies, including immunologic profiling, may help elucidate the mechanistic bases of these clinical differences.

## 5. Conclusions

This study demonstrated that patients with MPO-ANCA-positive EGPA fulfilling the 2022 ACR/EULAR classification criteria exhibited a greater disease burden and poorer health-related quality of life than patients with MPO-ANCA-negative EGPA, as reflected by a higher FFS, elevated markers of systemic inflammation, and lower SF-36 PCS scores. However, traditional distinctions between vasculitic and eosinophilic manifestations in ANCA-positive and -negative patients were not observed. Moreover, MPO-ANCA positivity was not significantly associated with poor outcomes, including all-cause mortality, relapse, and major organ complications such as ESKD, CVA, and ACS. These findings suggest that MPO-ANCA positivity in EGPA may be more closely linked to overall disease severity and quality of life than to the distinctions between vasculitic and eosinophilic manifestations or prognosis. Further studies are warranted to better delineate the clinical and biological implications of MPO-ANCA status in EGPA. By applying the updated 2022 ACR/EULAR classification criteria to a real-world cohort, our study offers new perspectives on the clinical burden of MPO-ANCA-positive EGPA and underscores the need for enhanced surveillance of this subgroup of patients.

## Figures and Tables

**Table 1:** Baseline characteristics of 57 patients with EGPA without PR3-ANCA.

Variables	Values
**Demographic data**	
Age (years)	53.0 (42.5–63.5)
Male sex (N, (%))	19 (33.3)
Body mass index (kg/m^2^)	22.6 (19.5–24.8)
Overweight ^a^ (N, (%))	9 (15.8)
Obese ^b^ (N, (%))	4 (7.0)
Ever smoker (N, (%))	3 (5.3)
**ANCA positivity (N, (%))**	
MPO-ANCA (or P-ANCA)-positive	25 (43.9)
PR3-ANCA (or C-ANCA)-positive	0 (0)
Both ANCA-positive	0 (0)
**AAV-specific indices**	
BVAS	11.0 (8.0–17.0)
FFS	1.0 (0–1.0)
SF-36 PCS	47.4 (31.1–65.4)
SF-36 MCS	54.1 (41.4–76.1)
**Manifestations based on 2022 ACR/EULAR EGPA classification criteria (N, (%))**	
Eosinophil count ≥ 1 × 10^9^/L (+5)	28 (49.1)
Obstructive airway disease (+3)	49 (86.0)
Nasal polyps (+3)	46 (80.7)
PR3-ANCA (or C-ANCA)-positive (−3)	0 (0)
Extravascular eosinophilic predominant inflammation (+2)	23 (40.4)
Mononeuritis multiplex/motor neuropathy (+1)	30 (52.6)
Haematuria (−1)	11 (19.3)
2022 ACR/EULAR classification criteria score	8.0 (7.0–11.5)
**Systemic manifestations based on BVAS (N, (%))**	
General	17 (29.8)
Cutaneous	17 (29.8)
Mucous and ocular	2 (3.5)
Otorhinolaryngological	47 (82.5)
Pulmonary	39 (68.4)
Cardiovascular	7 (12.3)
Gastrointestinal	3 (5.3)
Renal	13 (22.8)
Central and peripheral nervous systemic	34 (59.6)
**Acute phase reactants**	
ESR (mm/hr)	41.0 (10.5–68.0)
CRP (mg/L)	5.6 (1.1–22.0)
**Laboratory results**	
White blood cell count (/mm^3^)	10,330.0 (7050.0–14,020.0)
Eosinophil count (/mm^3^)	790.0 (300.0–4290.0)
Haemoglobin (g/dL)	13.4 (12.2–14.4)
Platelet count (×1000/mm^3^)	283.0 (231.5–379.5)
Fasting glucose (mg/dL)	102.0 (88.0–113.0)
Blood urea nitrogen (mg/dL)	13.5 (10.2–19.1)
Serum creatinine (mg/dL)	0.7 (0.6–0.9)
Total protein (g/dL)	6.8 (6.1–7.5)
Serum albumin (g/dL)	3.8 (3.4–4.3)
Total cholesterol (mg/dL)	174.0 (151.5–204.0)
HDL-C (mg/dL)	56.0 (41.5–70.5)
Triglyceride (mg/dL)	105.0 (79.5–165.0)
LDL-C (mg/dL)	90.0 (73.5–118.0)
**Comorbidities (N, (%))**	
Type 2 diabetes mellitus	9 (15.8)
Hypertension	18 (31.6)
Dyslipidaemia	8 (14.0)

Values are expressed as median (Q1–Q3 range) or N (%). ^a^ Overweight: 25 kg/m^2^ ≤ body mass index < 30 kg/m^2^. ^b^ Obese: 30 kg/m^2^ ≤ body mass index. EGPA: eosinophilic granulomatosis with polyangiitis; PR3: proteinase 3; ANCA: antineutrophil cytoplasmic antibody; MPO: myeloperoxidase; P: perinuclear; C: cytoplasmic; BVAS: Birmingham Vasculitis Activity Score; FFS: Five-Factor Score; SF-36: Short Form 36 health survey; PCS: Physical Component Summary; MCS: Mental Component Summary; ACR: American College of Rheumatology; EULAR: European Alliance of Associations for Rheumatology; ESR: erythrocyte sedimentation rate; CRP: C-reactive protein; HDL-C: high-density lipoprotein cholesterol; LDL-C: low-density lipoprotein cholesterol.

**Table 2:** Comparison of variables at diagnosis among patients with EGPA without PR3-ANCA according to MPO-ANCA positivity.

Variables	MPO-ANCA-Negative EGPA Without PR3-ANCA (N = 32)	MPO-ANCA-Positive EGPA Without PR3-ANCA (N = 25)	*p*-Value
**Demographic data**			
Age (years)	53.5 (39.3–58.5)	53.0 (44.5–67.5)	0.35
Male sex (N, (%))	12 (37.5)	7 (28.0)	0.45
Body mass index (kg/m^2^)	22.1 (19.4–24.7)	23.3 (19.5–25.1)	0.46
Overweight ^a^ (N, (%))	4 (12.5)	5 (20.0)	0.49
Obese ^b^ (N, (%))	3 (9.4)	1 (4.0)	0.62
Ever smoker (N, (%))	2 (6.3)	1 (4.0)	>0.99
**ANCA positivity (N, (%))**			
MPO-ANCA (or P-ANCA)-positive	0 (0)	25 (100)	**<0.001**
PR3-ANCA (or C-ANCA)-positive	0 (0)	0 (0)	NA
Both ANCA-positive	0 (0)	0 (0)	NA
**AAV-specific indices**			
BVAS	11.0 (7.0–15.8)	12.0 (9.0–19.5)	0.22
FFS	0 (0–1.0)	1.0 (0–1.0)	**0.038**
SF-36 PCS	52.5 (43.5–69.7)	35.0 (19.7–56.3)	**0.048**
SF-36 MCS	51.6 (43.0–82.8)	55.6 (36.6–63.5)	0.30
**Systemic manifestations (N, (%))**			
General	3 (9.4)	14 (56.0)	**<0.001**
Cutaneous	11 (34.4)	6 (24.0)	0.40
Mucous and ocular	1 (3.1)	1 (4.0)	>0.99
Otorhinolaryngological	27 (84.4)	20 (80.0)	0.67
Pulmonary	19 (59.4)	20 (80.0)	0.10
Cardiovascular	4 (12.5)	3 (12.0)	>0.99
Gastrointestinal	2 (6.3)	1 (4.0)	>0.99
Renal	5 (15.6)	8 (32.0)	0.14
Central and peripheral nervous systemic	17 (53.1)	17 (68.0)	0.26
**Acute phase reactants**			
ESR (mm/hr)	25.5 (7.0–63.8)	58.0 (16.0–97.5)	**0.026**
CRP (mg/L)	5.5 (0.9–11.6)	9.8 (1.4–83.9)	0.11
**Laboratory results**			
White blood cell count (/mm^3^)	8620.0 (6715.0–13,597.5)	12,660.0 (8730.0–15,600.0)	0.10
Eosinophil count (/mm^3^)	1100.0 (295.0–4730.0)	710.0 (280.0–4005.0)	0.73
Haemoglobin (g/dL)	13.7 (12.7–14.6)	12.7 (11.7–13.8)	**0.012**
Platelet count (× 1000/mm^3^)	263.0 (206.8–368.8)	327.0 (251.0–382.5)	0.19
Fasting glucose (mg/dL)	105.5 (91.3–113.5)	98.0 (85.5–113.0)	0.30
Blood urea nitrogen (mg/dL)	12.6 (9.1–17.8)	15.5 (12.1–23.6)	0.07
Serum creatinine (mg/dL)	0.8 (0.6–0.9)	0.7 (0.6–1.0)	0.76
Total protein (g/dL)	7.1 (6.3–7.5)	6.4 (6.1–7.3)	0.15
Serum albumin (g/dL)	4.1 (3.5–4.4)	3.7 (3.1–4.1)	**0.042**
Total cholesterol (mg/dL)	179.5 (145.8–210.0)	171.0 (162.0–191.0)	0.82
HDL-C (mg/dL)	54.0 (42.0–62.8)	65.0 (38.5–78.5)	0.36
Triglyceride (mg/dL)	103.5 (76.0–152.0)	117.0 (87.5–167.0)	0.45
LDL-C (mg/dL)	90.0 (75.5–127.5)	88.0 (73.0–115.5)	0.47
**Comorbidities (N, (%))**			
Type 2 diabetes mellitus	3 (9.4)	6 (24.0)	0.16
Hypertension	9 (28.1)	9 (36.0)	0.53
Dyslipidaemia	3 (9.4)	5 (20.0)	0.28

Values are expressed as median (Q1–Q3 range) or N (%). *p*-Value < 0.05 are shown in bold. ^a^ Overweight: 25 kg/m^2^ ≤ body mass index < 30 kg/m^2^. ^b^ Obese: 30 kg/m^2^ ≤ body mass index. EGPA: eosinophilic granulomatosis with polyangiitis; PR3: proteinase 3; ANCA: antineutrophil cytoplasmic antibody; MPO: myeloperoxidase; P: perinuclear; PR3: proteinase 3; C: cytoplasmic; BVAS: Birmingham Vasculitis Activity Score; FFS: Five-Factor Score; SF-36: Short Form 36 health survey; PCS: Physical Component Summary; MCS: Mental Component Summary; ESR: erythrocyte sedimentation rate; CRP: C-reactive protein; HDL-C: high-density lipoprotein cholesterol; LDL-C: low-density lipoprotein cholesterol.

**Table 3:** Comparison of medications administered during follow-up among patients with EGPA without PR3-ANCA according to MPO-ANCA positivity.

Variables	MPO-ANCA-Negative EGPA Without PR3-ANCA (N = 32)	MPO-ANCA-Positive EGPA Without PR3-ANCA (N = 25)	*p*-Value
**Medications (N, (%))**			
Glucocorticoids	31 (96.9)	25 (100)	>0.99
Cyclophosphamide	13 (40.6)	14 (56.0)	0.25
Rituximab	1 (3.1)	2 (8.0)	0.58
Mycophenolate mofetil	2 (6.3)	4 (16.0)	0.39
Azathioprine	16 (50.0)	15 (60.0)	0.45
Tacrolimus	0 (0)	2 (8.0)	0.19
Methotrexate	3 (9.4)	4 (16.0)	0.69

Values are expressed as N (%). EGPA: eosinophilic granulomatosis with polyangiitis; PR3: proteinase 3; ANCA: antineutrophil cytoplasmic antibody; MPO: myeloperoxidase.

**Table 4:** Comparison of outcomes among patients with EGPA without PR3-ANCA according to MPO-ANCA positivity.

Variables		MPO-ANCA-Negative EGPA Without PR3-ANCA (N = 32)	MPO-ANCA-Positive EGPA Without PR3-ANCA (N = 25)	*p*-Value
**Outcomes (N, (%))**	**Follow-up (months)**			
All-cause mortality	59.6 (28.6–110.5)	0 (0.0)	1 (4.0)	0.44
Relapse	49.2 (26.9–84.2)	3 (9.4)	4 (16.0)	0.69
ESKD	50.4 (27.6–92.8)	1 (3.1)	1 (4.0)	>0.99
CVA	59.6 (26.9–110.5)	0 (0.0)	1 (4.0)	0.44
ACS	50.4 (28.4–92.8)	1 (3.1)	2 (8.0)	0.58

Values are expressed as median (Q1–Q3 range) or N (%). EGPA: eosinophilic granulomatosis with polyangiitis; PR3: proteinase 3; ANCA: antineutrophil cytoplasmic antibody; MPO: myeloperoxidase; ESKD: end-stage kidney disease; CVA: cerebrovascular accident; ACS: acute coronary syndrome.

## Data Availability

The original contributions presented in this study are included in the article. Further enquiries can be directed to the corresponding author.

## Abbreviations

The following abbreviations are used in this manuscript:

AAV	anti-neutrophil cytoplasmic antibody-associated vasculitis
ACR	American College of Rheumatology
ACS	acute coronary syndrome
ANC	anti-neutrophil cytoplasmic antibody
BVA	Birmingham Vasculitis Activity Score
CRP	C-reactive protein
CVA	cerebrovascular accident
EGPA	eosinophilic granulomatosis with polyangiitis
ENT	ear, nose, and throat
ESKD	end-stage kidney disease
ESR	erythrocyte sedimentation rate
EULAR	European Alliance of Associations for Rheumatology
FFS	Five-Factor Score
MCS	Mental Component Summary
MPA	Microscopic polyangiitis
MPO	myeloperoxidase
PCS	Physical Component Summary
SF-36	Short Form 36
SHAVE	Severance Hospital ANCA-associated vasculitides
